# Tailoring of magnetoimpedance effect and magnetic softness of Fe-rich glass-coated microwires by stress- annealing

**DOI:** 10.1038/s41598-018-21356-3

**Published:** 2018-02-16

**Authors:** V. Zhukova, J. M. Blanco, M. Ipatov, M. Churyukanova, S. Taskaev, A. Zhukov

**Affiliations:** 1 Dpto. Fisica de Materiales, Fac. Quimicas, UPV/EHU, 20018 San Sebastian, Spain; 2 Dpto. de Física Aplicada, EUPDS, UPV/EHU, 20018 San Sebastian, Spain; 3 National University of Science and Technology «MISIS», Moscow, 119049 Russia; 4 NRU South Ural State University, Chelyabinsk, 454080 Russia; 5 IKERBASQUE, Basque Foundation for Science, 48011 Bilbao, Spain

## Abstract

There is a pressing need for improving of the high-frequency magneto-impedance effect of cost-effective soft magnetic materials for use in high-performance sensing devices. The impact of the stress-annealing on magnetic properties and high frequency impedance of Fe-rich glass-coated microwires was studied. Hysteresis loops of Fe-rich microwires have been considerably affected by stress- annealing. In stress-annealed Fe- rich microwire we obtained drastic decreasing of coercivity and change of character of hysteresis loop from rectangular to linear. By controlling stress-annealing conditions (temperature and time) we achieved drastic increasing (by order of magnitude) of giant magnetoimpedance ratio. Coercivity, remanent magnetization, diagonal and of-diagonal magnetoimpedance effect of Fe-rich microwires can be tuned by stress-annealing conditions: annealing temperature and time. Observed experimental results are discussed considering relaxation of internal stresses, compressive “back-stresses” arising after stress annealing and topological short range ordering.

## Introduction

Studies of magnetic wires have drawn considerable attention to the scientific community over the past few decades due to its immense possibilities for the development of low cost, highly sensitive magnetic sensors and devices^1–4^. Among the magnetic properties that considered as the most promising for industrial applications are the giant magnetoimpedance (GMI) as well as controlled domain wall (DW) propagation that can be realized in different families of magnetic wires^1–6^. In fact both GMI effect and DW propagation have been observed in crystalline Permalloy magnetic wires many years ago^3,4^. But magnetic properties of crystalline wires to great extend are affected by microstructure, i.e. by crystallographic defects, grain size, grain boundaries crystalline texture, and therefore require careful post-processing treatments for optimization of aforementioned properties^1,7^. Therefore amorphous materials prepared by rapid quenching from the melt characterized by the absence of long-range atomic order have attracted considerable attention since 70-s^1,2,8^.

Consequently various families of amorphous magnetic wires have been intensively studied along the last few decades^1,2,5–7,9^. Amorphous wires present a number of advantages, among them fast and inexpensive preparation method, excellent soft magnetic properties combined with good mechanical and corrosion characteristics^5–7,9,10^.

Since the last generation electronic devices increasingly demand more miniaturization, the use of thinner magnetic wires has special implications that involve increasingly smaller components^1,2,7^. Technological progress in rapid quenching techniques allowed development of a few novel methods suitable for preparation of micrometric and even sub-micrometric cast amorphous wires using either cold-drawn, melt extraction or glass-coated technologies^1,2,5–7,11–13^.

Actually there are a few families of amorphous magnetic wires that can be prepared using different fabrication processes involving rapid quenching from the melt:

(i) Conventional amorphous wires prepared using “in-rotating water” method (diameters of the order of 100 μm)^5,9^. The disadvantage of these wires is rather thick diameter. Therefore cold-drawn method is proposed for the diameter reduction^5^.
(ii) Melt extracted amorphous microwires (diameters of 30–60 μm) known since beginning of 90-th^11,14^. These microwires present not perfectly cylindrical shape that can affect the magnetic properties and hence GMI effect.
(iii) glass-coated microwires (with typical metallic nucleus diameters of 0.5–40 μm) prepared using so-called Taylor-Ulitovky method (also known as quenching-and-drawing method) known since 60-s^15,16^, but extensively studied starting from 90-s^5,7,12,13^. This fabrication method involves rapid quenching from the melt of perfectly cylindrical metallic alloy nucleus surrounded by glass-coating. The characteristic feature of these microwires is the enhanced magnetoelastic anisotropy arising by rapid quenching itself as well by the difference in thermal expansion coefficients^17–19^.

Among the properties of amorphous wires the GMI effect is actually one of the most attractive phenomena suitable for a number of technological applications such as magnetic sensors, memories and devices, smart composites for remote stress and temperature monitoring, health monitoring etc^20–25^. The main reason for elevated interest in GMI effect is related to the high sensitivity of the impedance to an applied magnetic field achieving up to 600% relative change of impedance of soft magnetic wires allowing detection of extremely low magnetic field.

Usually magnetic field dependences of impedance, *Z*, is expressed through the GMI ratio, *ΔZ/Z*, defined as:

1 $${\rm{\Delta }}Z/Z=[Z(H)-Z({H}_{max})]/Z({H}_{max}),$$

where *H*_*max*_ is the maximum applied DC magnetic field.

Reported magnetic field sensitivity (up to 10%/A/m) of GMI effect in amorphous wires is one of the largest among the non-cryogenic effects^26,27^. It is worth mentioning that the theoretical maximum GMI ratio is about 3000% being few times larger than the GMI ratios reported up to now^28^. Moreover theoretical minimum skin depth is about 0.3 μm^29^.

It is commonly accepted that the origin of GMI is related to the classical skin effect of magnetic conductor^30,31^.

Consequently the guidelines for searching of magnetic materials presenting the largest GMI effect are laying in design of soft magnets thicker than few μm (about one order thicker than the minimal skin depth) with low magnetic anisotropy.

Glass-coated microwires prepared using Taylor-Ulitovsky method with typical metallic nucleus diameter of a few μm is therefore one of the most promising materials for achievement of the largest GMI effect. Up to now the highest GMI ratio is reported for glass-coated microwires with nearly-zero Co-rich microwires with diameters of the order of a few μm^26,27^. But for some industrial applications (i.e. tunable metamaterials for electromagnetic cloaking, imaging, stress and temperature monitoring containing the microwires inclusions in the dielectric matrix or large scale production of magnetic sensors) involving GMI effect a large amount of magnetic wires can be required. Therefore the development of cost-effective magnetically soft microwires is highly demanded for prospective applications^1,6^.

Less expensive Fe-rich amorphous glass-coated microwires are the good candidates, but usually highly magnetostrictive as-prepared Fe-rich amorphous glass-coated microwires present low circumferential magnetic permeability and therefore low GMI effect^6,12,32^. As reported elsewhere^12,32^ Fe-rich microwires amorphous microwires with positive magnetostriction coefficient usually present rectangular hysteresis loop related to a domain structure consisting of a large axially magnetized single domain surrounded by outer domains with radial magnetization orientation^6,12^. Such domain structure is related to magnetoelastic anisotropy, i.e. high internal stresses and magnetostriction coefficient. Consequently enhancement of magnetic softness and GMI effect of Finemet-type Fe-rich microwires by nanocrystallization allowing reduction of the magnetostriction coefficient has been reported^33,34^. But Finemet-type nanocrystalline materials are rather brittle.

Induced magnetic anisotropy is the alternative route for optimization of magnetic softness of amorphous microwires^35–37^. The principal advantage of stress-induced anisotropy is that it allows maintaining superior mechanical properties typical for amorphous materials. Previously change of hysteresis loop and increasing of GMI ratio at low-frequencies (10 MHz) are reported in stress-annealed Fe_69_B_12_Si_14_C_5_ glass-coated microwires^35^. Influence of induced magnetic anisotropy on high frequency (above 100 MHz) GMI effect in stress- annealed Fe-rich microwires is less studied. Only a few recent publications report on improvement of GMI ratio in stress-annealed Co-rich and Fe-rich amorphous microwires^36,37^. It is worth mentioning that the diameter reduction achieved in microwires with metallic nucleus diameter of a few μm must be associated with the shift of the optimal GMI frequency range towards higher frequencies^38^. Therefore for magnetic microwires the optimal GMI frequency range of the order of 100–500 MHz is reported elsewhere^39^.

Consequently, in this paper we present our recent experimental results on effect of stress-annealing on magnetic properties and high frequency GMI effect of Fe-rich glass-coated microwires.

## Results and Discussion

**Figure 1:**
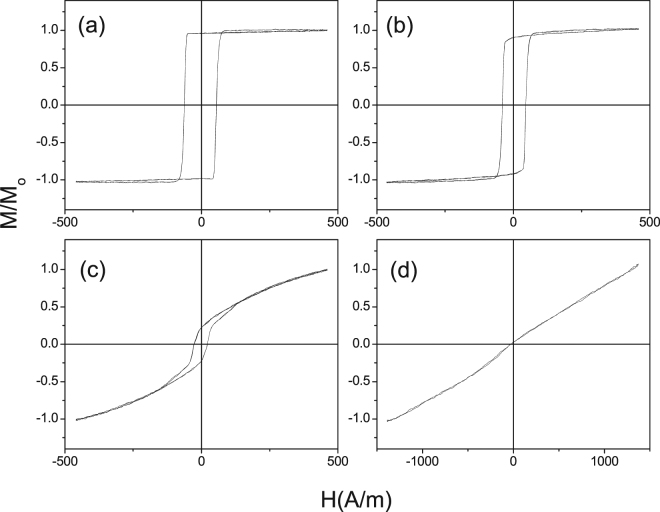
Hysteresis loops as-prepared (**a**), annealed at 200 °C (**b**), 250 °C (**c**) and 300 °C (**d**) for 1 h Fe_75_B_9_Si_12_C_4_ microwires.

As expected from previous knowledge on Fe-rich microwires^40^ as-prepared Fe_75_B_9_Si_12_C_4_ microwires present perfectly rectangular hysteresis loops (Fig. 1).

Increasing the annealing temperature, *T*_*ann*_, and keeping the same annealing time (*t*_*ann*_ = 1 h) we observed drastic change of the hysteresis loops from perfectly rectangular to linear with quite low coercivity (see Fig. 1a–d).

**Figure 2:**
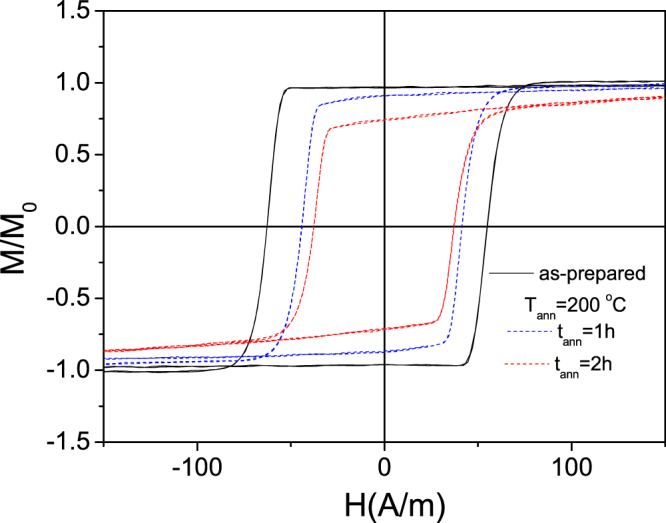
Hysteresis loops of as-prepared and stress-annealed at *T*_*ann*_ = 200 °C for different *t*_*ann*_ measured for Fe_75_B_9_Si_12_C_4_ microwires.

Additionally, fixing *T*_*ann*_ and rising annealing time we observed similar tendency: decreasing of coercivity, *H*_*c*_, and squireness ratio, *M*_*r*_*/M*_*s*_ (Fig. 2).

Observed changes must be associated with changes of magnetic anisotropy and domain structure after stress- annealing.

The hysteresis loops of stress-annealed Fe-rich microwires are becoming similar that of Co-rich microwires in which the remagnetization process in axial direction is associated to the magnetization rotation^27^. Additionally, observed transversal magnetic anisotropy can be tuned by stress-annealing conditions (time and temperature, see Figs 1, 2).

As pointed out previously from direct and indirect experiments^6,41,42^ the domain structure of magnetic wires is usually described as consisting of a large axially magnetized single domain surrounded by outer domains. Moreover the radius of the inner axially magnetized core radius, *R*_*ic*_, can be estimated from squireness ratio, *M*_*r*_*/M*_*s*_ as:^41^

2 $${R}_{ic}=R{({M}_{r}/{M}_{s})}^{l/2},$$

being *R*- microwire radius.

**Figure 3:**
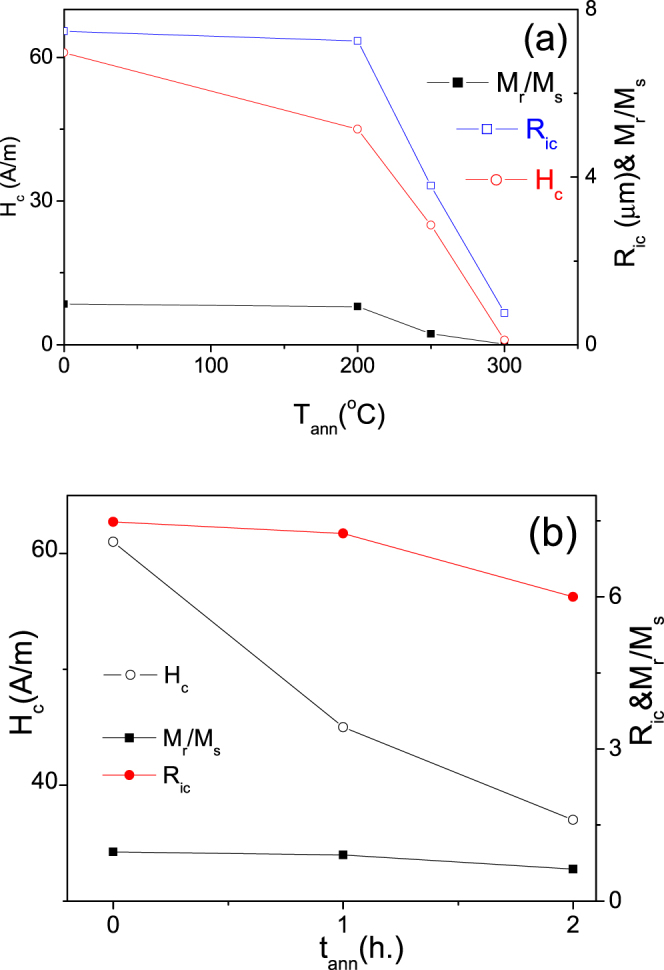
Dependence of coercivity, *H*_*c*_, squireness ratio, *M*_*r*_*/M*_*s*_ and radius of inner axially magnetized core, *R*_*ic*_, on annealing temperature (**a**) and annealing time (**b**) measured for Fe_75_B_9_Si_12_C_4_ microwires

As can be observed from Fig. 3a *M*_*r*_*/M*_*s*_ –ratio and H_c_ decrease rising the stress- annealing temperature (Fig. 3a) and time (Fig. 3b).

Consequently, after stress- annealing *R*_*i*c_ decreases achieving 0.1 *R* at *T*_*ann*_ = 300 °C. Therefore we must assume that the inner axially magnetized core radius, *R*_*c*_, decreases after stress- annealing as shown in Fig. 3a,b.

One can expect that observed stress-induced anisotropy must affect GMI effect of studied microwires. Indeed, theoretically and experimentally shown elsewhere^43–45^ that easy magnetic anisotropy direction and magnetic anisotropy field affect both value and magnetic field dependence of GMI effect of magnetic wires and magnetic softness of amorphous wire is one of the most important conditions to observe high GMI effect.

Consequently, we measured GMI effect in as-prepared and stress-annealed Fe_75_B_9_Si_12_C_4_ microwires.

**Figure 4:**
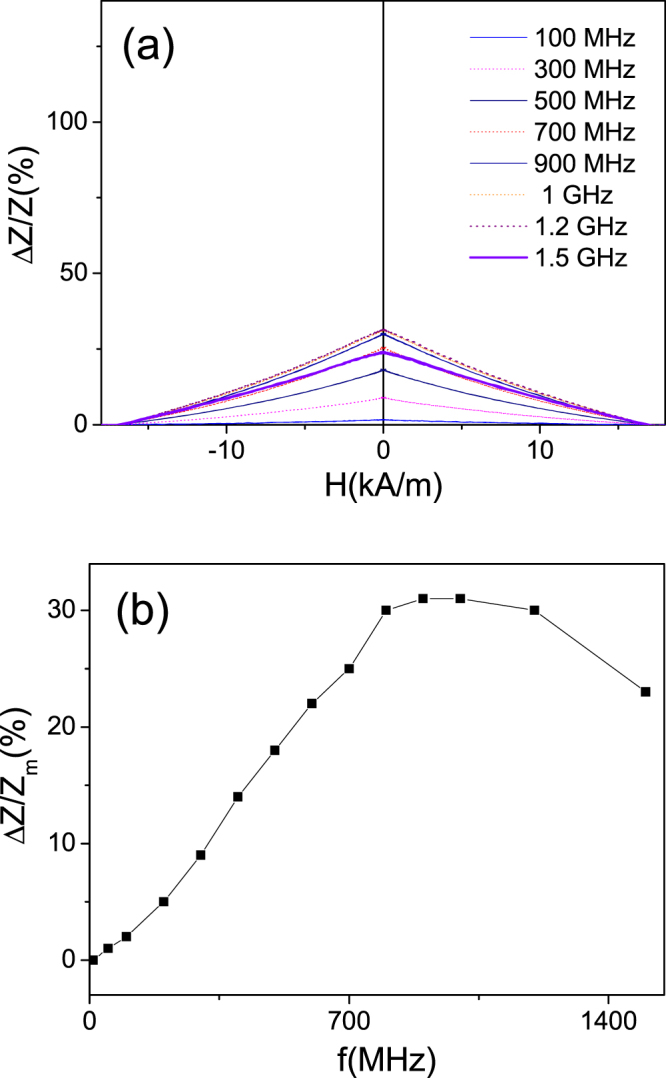
Δ*Z/Z(H)* dependencies measured in as-prepared Fe_75_B_9_Si_12_C_4_ microwires at different frequencies (**a**) and frequency dependence of maximum GMI ratio (**b**)

As expected from previous knowledge on GMI effect of Fe-rich microwires with axial magnetic anisotropy, as-prepared Fe_75_B_9_Si_12_C_4_ microwires present rather poor GMI effect (Fig. 4a): at low frequencies (about 10 MHz, i.e where most of experimental results are reported) the GMI ratio is almost negligible. Rising the frequency, *f*, we observed some increasing of the GMI ratio (Fig. 4a,b). At about *f* = 800 MHz maximum GMI ratio, ΔZ/Z_m_ achieves 30% (Fig. 4b). Increasing the frequency above 1 GHz a decreasing of ΔZ/Z_m_ is observed.

As commonly accepted elsewhere^2,38,46^ at f ≤ 10 MHz the GMI effect is basically related to variations of the magnetic penetration depth due to strong changes of the effective magnetic permeability caused by a dc magnetic field^2,30,31,39,46^ associated to both domain-wall movement and magnetization rotation.

For higher frequencies (up to GHz) the GMI effect is also originated by the skin effect of the magnetic conductor, but the domain walls are strongly damped. Consequently, the magnetization rotation is assumed responsible for the GMI effect^2,39,46^. At GHz frequencies, the GMI presents features similar to the ferromagnetic resonance (FMR)^2,7,39,46^.

Analysis of the magnetic field dependencies of GMI ratio can provide insight into the effect of stress-annealing on magnetic anisotropy and domain structure of Fe-rich microwires.

It is worth mentioning that GMI effect of as-prepared Fe_75_B_9_Si_12_C_4_ microwires present features typical for magnetic wires with axial magnetic anisotropy, i.e decay with increasing of magnetic field (see Fig. 4a).

**Figure 5:**
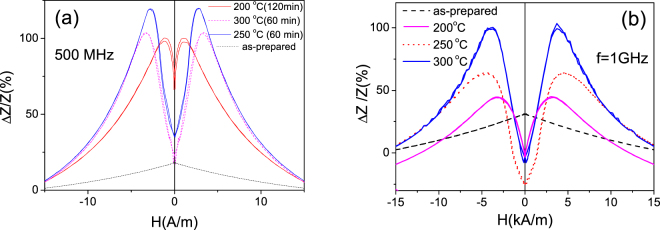
Δ*Z/Z(H)* dependences measured in as-prepared and stress-annealed at different *T*_*ann*_ in Fe_75_B_9_Si_12_C_4_ microwires measured at 500 MHz (**a**) and1 GHz (**b**)

Stress-annealed Fe_75_B_9_Si_12_C_4_ microwires present rather different value and magnetic field dependence of GMI ratio: all stress-annealed Fe_75_B_9_Si_12_C_4_ microwires present double-peak ΔZ/Z(H) dependencies and higher *ΔZ/Z*_*m*_ -values (see Fig. 5a,b). As mentioned above, such double-peak *ΔZ/Z(H)* dependencies are predicted for magnetic wires with circumferential magnetic anisotropy^43,45^.

As can be appreciated from Fig. 5a,b, GMI ratio in Fe_75_B_9_Si_12_C_4_ microwires stress-annealed at all studied conditions (*T*_*ann*_) is almost one order of magnitude higher than in as-prepared Fe_75_B_9_Si_12_C_4_ microwires. Below we present more detailed studies of GMI effect in stress-annealed Fe_75_B_9_Si_12_C_4_ microwires.

**Figure 6:**
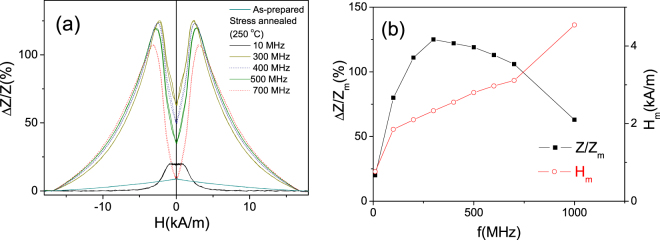
*ΔZ/Z(H)* dependencies measured at various *f*-values (**a**) and *ΔZ/Z*_*m*_*(f)* and *H*_*m*_*(f)* dependencies in stress- annealed at T_ann_ = 250 °C for *t*_*ann*_ = 60 min Fe_75_B_9_Si_12_C_4_ microwires

A significant enhancement of the GMI effect at all frequencies is observed for Fe_75_B_9_Si_12_C_4_ microwire stress-annealed at *T*_*ann*_ = 250 °C for *t*_*ann*_ = 60 min (see Fig. 6). The most noticeable are the enhanced *ΔZ/Z*_*m*_ -values observed for the frequency band of about 300 MHz, where more than one order increasing of the GMI ratio (up to *ΔZ/Z*_*m*_ ≈ 125%) by stress-annealing is achieved (compare Figs 4b and 6b).

**Figure 7:**
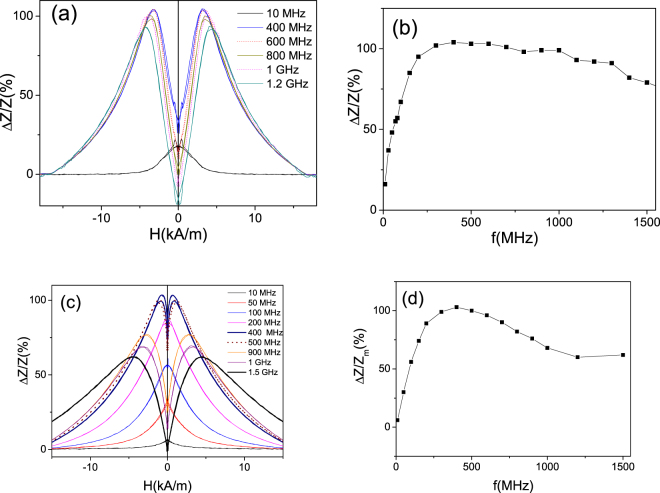
Δ*Z/Z(H)* dependencies measured at various *f*-values (**a**,**c**) and *ΔZ/Z*_*m*_*(f)* dependencies (**b**,**d**) in stress- annealed at T_ann_ = 300 °C for *t*_*ann*_ = 60 min (**a**,**b**) and at T_ann_ = 200 °C for *t*_*ann*_ = 120 min (**c**,**d**) Fe_75_B_9_Si_12_C_4_ microwires.

Similarly, a beneficial increase of GMI ratio is observed for Fe_75_B_9_Si_12_C_4_ microwire stress-annealed at *T*_*ann*_ = 300 °C for *t*_*ann*_ = 60 min and at *T*_*ann*_ = 200 °C for *t*_*ann*_ = 120 min (see Fig. 7). In the sample stress-annealed at *T*_*ann*_ = 300 °C for *t*_*ann*_ = 60 min GMI ratio values of about 100% are observed in a wide frequency band from 200 MHz up to 1 GHz (Fig. 7a,b).

Considerable increasing of GMI ratio is observed for Fe_75_B_9_Si_12_C_4_ microwire stress-annealed at *T*_*ann*_ = 200 °C for *t*_*ann*_ = 120 min (see Fig. 7 c,d). At these annealing conditions again *ΔZ/Z*_*m*_ ≈ 100% is observed for frequencies about 400–600 MHz (Fig. 7d).

**Figure 8:**
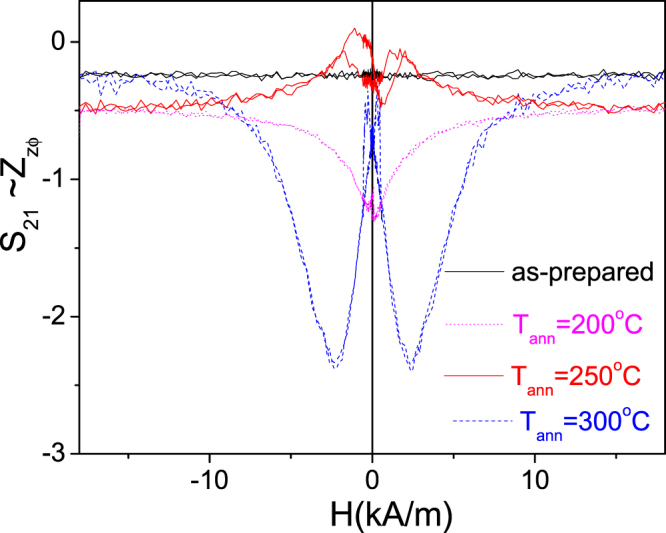
*S*_21_*(H)* dependencies measured in as-prepared and stress-annealed at different *T*_*ann*_ Fe_75_B_9_Si_12_C_4_ microwires.

In as-prepared Fe_75_B_9_Si_12_C_4_ microwire the off-diagonal *Z*_*zφ*_ components (*S*_*21*_–values) present nearly- zero values. Stress-annealing had a beneficial effect on the off-diagonal MI effect, *S*_*21*_, as shown in Fig. 8. For all annealed conditions (*T*_*ann*_) we observed considerable increasing of *S*_*21*_–values.

**Figure 9:**
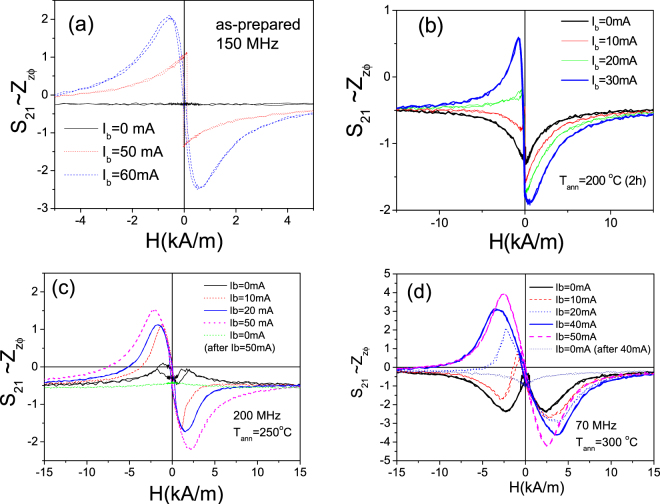
*S*_21_*(H)* dependencies measured in as-prepared (**a**) and stress-annealed at T_ann_ = 200 °C for *t*_*ann*_ = 120 min (**b**) T_ann_ = 250 °C for *t*_*ann*_ = 60 min (**c**) and T_ann_ = 300 °C for *t*_*ann*_ = 60 min (**d**) Fe_75_B_9_Si_12_C_4_ microwires.

Additionally, we observed increasing of S_21_-values upon application of the bias current, *I*_*b*_, in as-prepared and stress-annealed Fe_75_B_9_Si_12_C_4_ sample (see Fig. 9).

The highest S_21_-values (up to 4%) upon application of the bias current were observed in Fe_75_B_9_Si_12_C_4_ sample stress-annealed at 300 °C.

All stress- annealed samples exhibit double-peak shape ΔZ/Z (H) dependencies (see Figs 6a,7a and c) that suggest existence of circular magnetic anisotropy. At *I*_*b*_ = 0, the off- diagonal impedance is low and irregular. In nearly-zero magnetostricvite microwires with spontaneous circular magnetic anisotropy such behavior is associated to a bamboo-like domain structure of the outer domain shell^46^. As mentioned above application of bias current makes the off-diagonal MI higher with characteristic asymmetric dependence on magnetic field (see Fig. 9). This dependence of *S*_21_ on *I*_*b*_ can be interpreted as growth of the domains with magnetization parallel to the circular field, *H*_*b*_, at the expense of domains with magnetization antiparallel to *H*_*b*_^47^.

On the other hand observed S_21_*-* values (with maximum up to 4% for the sample stress-annealed at T_ann_ = 300 °C for *t*_*ann*_ = 60 min, see Fig. 9d) are lower than that observed in the nearly- zero magnetostrictive microwire where *S*_*21*_*-* values can reach 15%^47,48^. Additionally under application of *I*_*b*_ = 7–10 mA we observed transformation of the bamboo-like domain structure into a single domain in nearly- zero magnetostrictive (Co-rich) microwires^48^. Studied Fe-rich microwires present rather high (about 35 × 10^−6^) values of the magnetostriction coefficient and therefore elevated magnetoelastic anisotropy^49^. Therefore we can assume that the applied circular magnetic field *H*_*b*_ (associated to bias current *I*_*b*_) is not enough to remove the bamboo-like surface domain structure.

**Figure 10:**
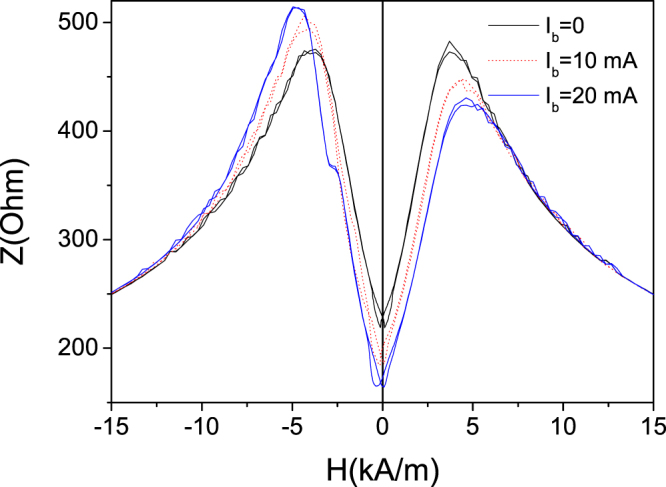
*Z(H)* dependencies measured in stress-annealed (*T*_*ann*_ = *300 *°C) Fe_75_B_9_Si_12_C_4_ microwires at different *I*_*b*_-values.

It is worth mentioning that when the bias current is applied, the impedance, *Z*, dependence on magnetic field becomes asymmetric (see Fig. 10) that suggests the existence of a helical anisotropy^48^.

There is an evidence that elevated values of DC current flowing through the sample can produce Joule heating of the samples. When we applied a higher current (*I*_*b*_ = 40-50 mA), the samples are heated due to Joule effect before the required current to remove the domains structure is reached. The irreversible changes of *S*_*21*_-values can be appreciated after application of *I*_*b*_ = 40-50 mA (see Fig. 9). Indeed for *I*_*b*_ = 50 mA the current density, *j*, is estimated as *j* ≈ 300 A/mm^2^. Earlier magnetic hardening and/or crystallization of the microwires after annealing with the DC current density, *j* ≈ 450 A/mm^2^ was reported^45,50^.

**Figure 11:**
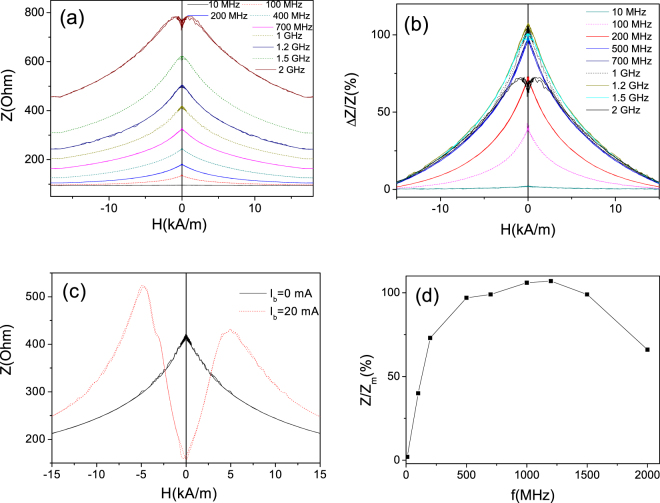
*Z(H)* dependencies (**a**), *ΔZ*/*Z(H)* dependencies (**b**) measured at different *f*-values, influence of bias current on Z(H) dependencies measured at 1 GHz (**c**) and *ΔZ/Z*_*m*_*(f)* dependence (**d**) observed in Joule heated at 40 mA stress-annealed (*T*_*ann*_* = 300* °C) Fe_75_B_9_Si_12_C_4_ microwires.

Observed Joule heating of stress-annealed Fe_75_B_9_Si_12_C_4_ microwires can considerably affect diagonal MI effect in stress-annealed samples providing interesting features. Particularly after Joule heating at 40 mA of stress-annealed at *T*_*ann*_ = 300 °C sample GMI ratio is still rather high (above 100%), but magnetic field dependence of impedance, *Z*, and Δ*Z/Z(H)* dependence present monotonic decay for a wide frequencies range (see Fig. 11 a,b). Additionally application of bias current (*I*_*b*_ = 20 mA) produces switching from single peak to double peak *Z(H)* dependence (Fig. 11c). Such considerable effect of bias current on *Z(H)* dependence and single peak Δ*Z/Z(H)* dependence must be attributed to quite low axial magnetic anisotropy in Joule heated after stress-annealing at *T*_*ann*_ = 300 °C sample. The application of bias current must be associated to the circular magnetic field (Oersted field) given in the surface of the metallic nucleus by formula:

3 $${H}_{circ}=I/2\pi r$$

where *I* is the current value, *r*- radial distance.

This Oersted circular magnetic field can therefore switch the magnetization from axial to circular orientation in the surface layer of metallic nucleus.

Observed Δ*Z/Z*_*m*_*(f)* dependence presents quite wide optimal frequency range (from 500 MHz up to 1.5 GHz) at which Δ*Z/Z*_*m*_ ≈ 100% (Fig. 11d).

**Figure 12:**
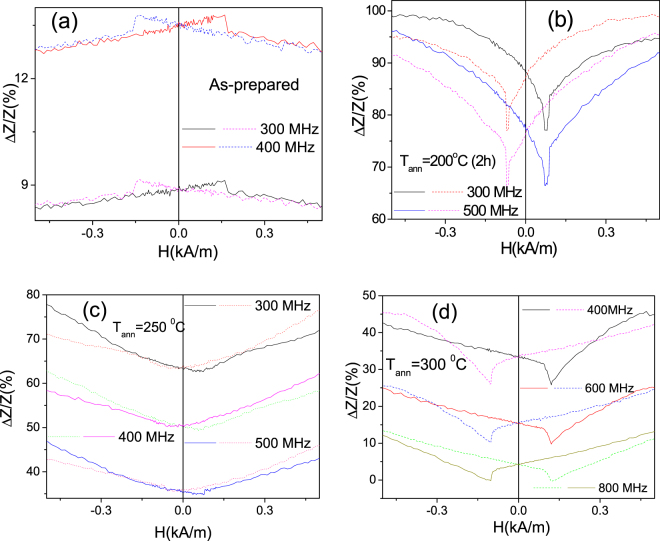
GMI hysteresis observed in (**a**) as-prepared, stress-annealed at *T*_*ann*_* = 200* °C (**b**), *T*_*ann*_* = 250* °C (**c**) *T*_*ann*_* = 300* °C (**d**) Fe_75_B_9_Si_12_C_4_ microwire.

The other common feature of GMI effect in all studied (as-prepared and stress-annealed at all temperatures) is the low field GMI hysteresis (see Fig. 12). At each frequency we provided *ΔZ*/*Z(H)* dependencies with ascending and descending magnetic field in order to illustrate the low field GMI hysteresis previously reported only for microwires with vanishing magnetostriction coefficient. Observed GMI hysteresis is independent on frequency and presents features similar to that previously reported for nearly-zero magnetostictive microwires^29,51,52^.

The origin of GMI hysteresis observed in nearly-zero magnetostictive magnetic wires has been discussed considering the deviation of the magnetic anisotropy easy axis form circumferential direction, the magnetostatic interaction of the inner axially magnetized core with the outer domain shell and the irreversible switches of the transverse permeability, caused by domain wall structure transitions^29,51,52^. Negligible frequency dependence (or even lack of dependence) of GMI hysteresis in our opinion can be attributed to the static remagnetization process of studied microwires.

**Figure 13:**
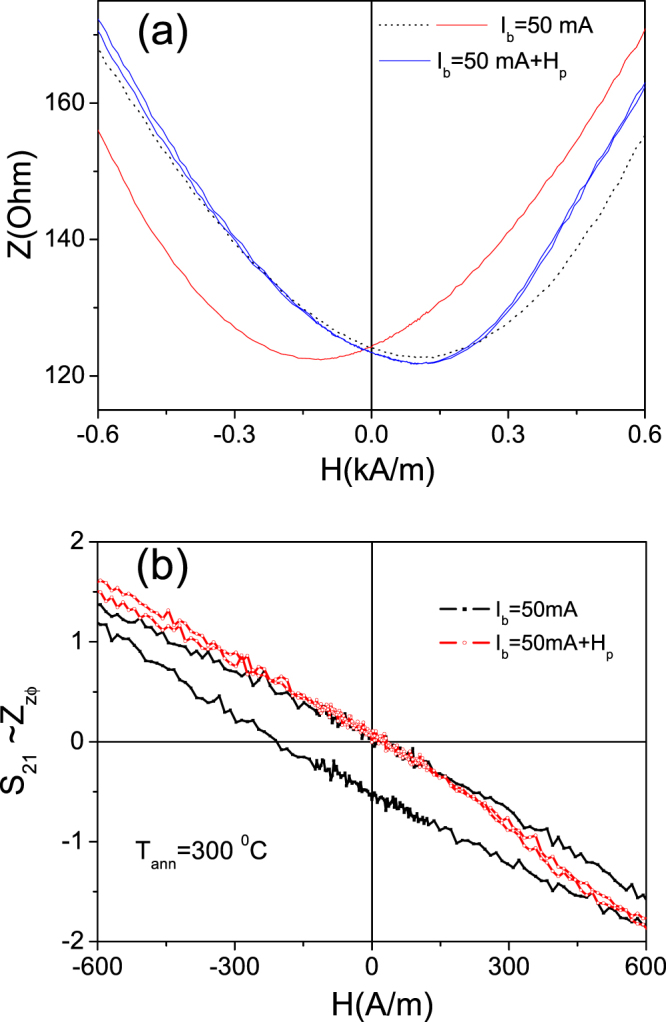
Effect of pulsed field on diagonal (**a**) and off-diagonal (**b**) GMI hysteresis observed in stress-annealed at *T*_*ann*_* = 3*00 °C Fe_75_B_9_Si_12_C_4_ microwire.

This assumption is confirmed by the influence of strong enough pulsed magnetic field (18 kA/m) applied before taking each measurement point on GMI hysteresis. As can be observed from Fig. 13, GMI hysteresis (diagonal and off-diagonal) can be suppressed by application of a pulsed magnetic field. Such influence has been previously interpreted^52^ considering that high enough (18 kA/m) applied magnetic field saturates the inner core. On the other hand in some cases the GMI hysteresis observed in studied Fe-rich microwires presents features (i.e. impedance jumps) similar to that reported for Co-rich with helical magnetic anisotropy in the outer domain shell^29^.

Considerable enhancement of ΔZ/Z and *S*_*21*_–values observed in stress-annealed Fe-rich microwires must be attributed to transverse magnetic anisotropy evidenced from comparison of hysteresis loops of as-prepared and stress –annealed microwires.

Rectangular hysteresis loop observed in as-prepared Fe-rich microwires (Fig. 1a) with positive magnetostriction coefficient is commonly attributed to the axial magnetic anisotropy related to the magnetoelastic anisotropy^17–19,40^. Indeed, the axial internal stresses in glass-coated microwires arising during the preparation process are the highest within the most part of the metallic nucleus^17,18,53^.

From previous studies is known that stresses and/or magnetic field annealing considerably affects magnetic anisotropy of amorphous materials^54,55^.

In particular annealing at temperatures below the Curie temperature can originate a macroscopic magnetic anisotropy with a preferred magnetization direction determined by the magnetization distribution during the annealing^54,56^. Such induced magnetic anisotropy depends on the annealing temperature, stress and magnetic applied during the annealing. Consequently macroscopically isotropic amorphous materials annealed at certain conditions (at the presence of magnetic field or stress) can exhibit macroscopic magnetic anisotropy.

The origin of field-induced anisotropy of amorphous materials has been discussed in terms of the directional ordering of atomic pairs or compositional short- range ordering^46,54^, although the topological short range ordering can play an important role^55^. Aforementioned topological short range ordering (also known as structural anisotropy) involves the angular distribution of the atomic bonds^55^ and small anisotropic structural rearrangements at temperature near the glass transition temperature^57^.

Mentioned pair ordering is commonly considered for amorphous alloys containing at least two magnetic elements^54–58^. Consequently for the studied Fe_75_B_9_Si_12_C_4_ amorphous microwires containing only one magnetic element (Fe) the pair ordering and the compositional short- range ordering mechanisms of stress- induced magnetic anisotropy must be disregarded.

The other approach involving the cluster model has been proposed for explaining the evolution of physical properties of amorphous materials under annealing^59^. However conventional furnace annealing at temperatures below the crystallization (generally below 500 °C) does not affect the character of hysteresis loop of Fe-rich glass-coated microwires^33,49^.

The case of glass-coated microwires is different: the presence of the glass-coating is associated to strong internal stresses. Previously the origin of stress-induced anisotropy in Fe-rich (Fe_74_B_13_Si_11_C_2_) amorphous microwires is discussed considering “back stresses” giving rise to the redistribution of the internal stresses after stress-annealing^35,36^.

In the present case the sample was heated, annealed and slowly cooled with the furnace under the applied tensile stress. Consequently observed transversal magnetic anisotropy can be explained considering either increasing of transversal anisotropy at the expense of axial anisotropy due to back stresses or aforementioned topological short range ordering.

The advantage of described above effective approach allowing improvement of magnetic softness and high frequency GMI effect of Fe-rich microwires is that proposed stress-annealing allows retain superior mechanical properties of amorphous materials, i.e. plasticity and flexibility.

## Methods

We studied the influence of stress-annealing on magnetic properties and GMI effect of Fe_75_B_9_Si_12_C_4_ amorphous glass-coated microwires (total diameter, D = 17.2 μm, metallic nucleus diameter, d = 15.2 μm) prepared by Taylor-Ulitovky method previously described elsewhere^7,15^.

**Figure 14:**
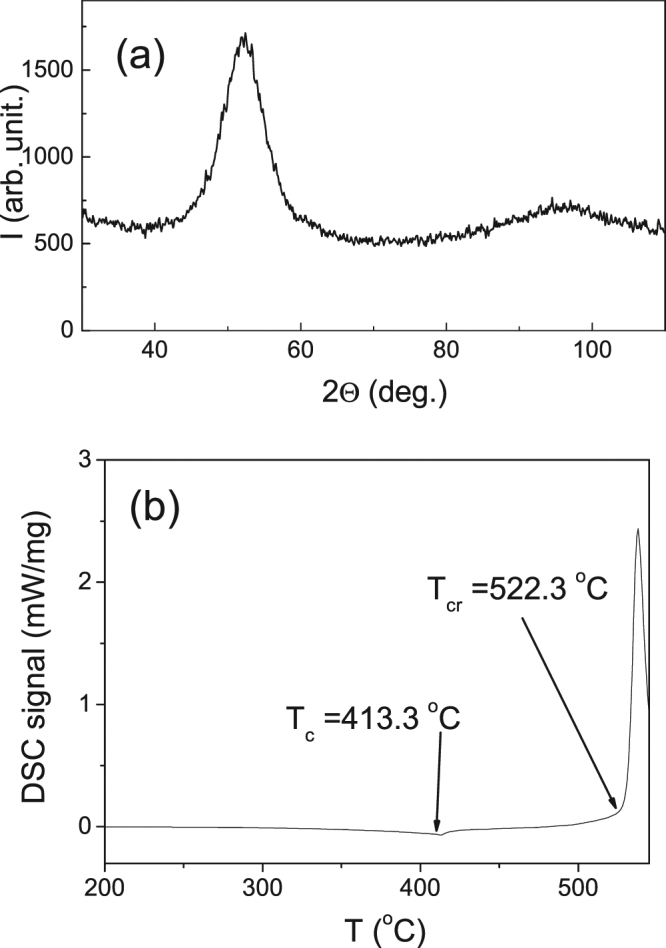
XRD (**a**) and DSC curves (**b**) of as-prepared Fe_75_B_9_Si_12_C_4_ microwire.

Structure of studied microwires has been checked by X-ray diffraction (XRD) using a BRUKER (D8 Advance) X-ray diffractometer with Cu K_α_ (λ = 1.54 Å) radiation. All as-prepared and annealed microwires present typical for amorphous alloys XRD patterns with broad halo (Fig. 14a). The crystallization, *T*_*cr*_, and Curie, *T*_*c*_, temperatures were determined using differential scanning calorimetry (DSC) measurements performed using DSC 204 F1 Netzsch calorimeter in Ar atmosphere at a heating rate of 10 K/min.

*T*_*cr*_ is determined as the beginning of the first crystallization peak. As can be seen from Fig. 14b in as-prepared Fe_75_B_9_Si_12_C_4_ microwire *T*_*cr1*_ ≈ 522 °C and *T*_*c*_ ≈ 413 °C.

Samples annealing has been performed in a conventional furnace at temperatures, *T*_*ann*_, below the crystallization temperature, *T*_*cr1*_, and Curie temperature, *T*_*c*_ (*T*_*ann*_ ≤ 300 °C). All the thermal treatments were perfomed in air because metallic nucleus are coated by the insulating and continuous glass coating.

The microwire was heated, annealed and slowly cooled with the furnace under the tensile stress.

This annealing is designed in order to avoid the influence of the stresses arising during the sample cooling.

The value of stresses applied during the heat treatment within the metallic nucleus and glass cover have been estimated as previously described elsewhere^36^:

4 $${\sigma }_{m}=\frac{K.P}{\,K\,{S}_{m}+{S}_{gl}},{\sigma }_{gl}=\frac{P}{\,K\,{S}_{m}+{S}_{gl}}$$

where *k = E*_2_/*E*_1_, *E*_2_ is the Young modulus of the metal, *E*_1_ – Young modulus of the glass at room temperature, *P* is the mechanical load applied during the annealing, and *S*_m_ and *S*_gl_ are the cross sections of the metallic nucleus and glass coating respectively. The estimated values of applied stress estimated using eq. (4) is σ_m_ ≈ 900 MPa.

We measured the hysteresis loops using fluxmetric method previously successfully employed by us for characterization of magnetically soft microwires^40^. The hysteresis loops are plotted as the dependence of normalized magnetization, *M/M*_*0*_ (where *M* is the sample´s magnetic moment at given magnetic field, H, and *M*_*0*_ is the sample’s magnetic moment at the maximum magnetic field amplitude, *H*_*m*_) on magnetic field, *H*.

For evaluation of the GMI effect we employed micro-strip sample holder previously described elsewhere^25,29^. A magnetic field, *H*, is produced by a long solenoid. The microwire impedance, Z, was evaluated from the reflection coefficient *S*_*11*_ measured by the vector network analyzer using the expression^25,29^:

5 $$Z={Z}_{0}(1+{S}_{11})(1-{S}_{11}),$$

where *Z*_0_ = 50 Ohm is the characteristic impedance of the coaxial line. The off-diagonal *Z*_*zφ*_ component has been evaluated from the transmission coefficient, *S*_21_^25,29^.

The GMI ratio, *ΔZ/Z*, is defined using eq. (1).

The magnetostriction coefficient of studied microwires has been evaluated using the small angle magnetization rotation method (SAMR) method^60^. Although initially this method was developed for amorphous materials in which the magnetization rotation presents the determining role^60^, recently we demonstrated the possibility to extend the SAMR method for the case of Fe-rich microwires presenting important contribution of domain wall propagation and designed a novel set-up for SAMR measurements^49^.

Using this method we estimated the *λ*_*s*_ –values for as-prepared and annealed samples. Evaluated *λ*_*s*_ -values of as-prepared Fe_75_B_9_Si_12_C_4_ samples are about 35 × 10^−6^ (similar to *λ*_*s*_ –values reported for Fe-rich amorphous materials^61,62^). We observed slight increasing of *λ*_*s*_ –values after stress-annealing (from 35 × 10^−6^ to 38 × 10^−6^). Although observed increase of *λ*_*s*_ –values is negligible it can be explained considering the stress dependence of the magnetostriction coefficient^62^. Indeed stress-relaxation associated to the annealing and compensation of the internal stresses by back stresses may originate the magnetostriction coefficient increasing.

## Conclusions

We have demonstrated an effective approach to improving the high frequency GMI effect and magnetic softness of Fe-rich microwires using stress-annealing. We found that diagonal and off-diagonal GMI effect and hysteresis loop of Fe-rich microwires are affected by stress-annealing conditions. We observed transformation of rectangular hysteresis loops to linear and beneficial increasing of diagonal MI effect by order of magnitude as well as increasing of the off-diagonal MI effect after stress-annealing for Fe-rich microwires. Stress-annealed Fe-rich microwires present high GMI ratio (above 100%) in extended frequency range (from 500 MHz up to 1.5 GHz). Additionally, GMI hysteresis is observed in as-prepared and stress-annealed Fe-rich microwires. Similarly to the case of nearly-zero magnetostrictive microwires we observed the GMI hysteresis that is almost independent on frequency and can be suppressed by application of a pulsed magnetic field.

Stress-annealed microwires present unusual features, like switching from single peak to double peak *Z(H)* dependence under application of bias current.

Observed stress -induced magnetic anisotropy of Fe-rich microwires is discussed considering increasing of transversal anisotropy at the expense of axial anisotropy due to back stresses and topological short range ordering.
